# An essential Aurora-related kinase transiently associates with spindle pole bodies during *Plasmodium falciparum* erythrocytic schizogony

**DOI:** 10.1111/j.1365-2958.2010.07442.x

**Published:** 2011-01

**Authors:** Luc Reininger, Jonathan M Wilkes, Hélène Bourgade, Diego Miranda-Saavedra, Christian Doerig

**Affiliations:** 1 INSERM-EPFL Joint Laboratory, Global Health InstituteEPFL-SV-GHI, Station 19, CH-1015 Lausanne, Switzerland; 2 Wellcome Trust Centre for Molecular Parasitology, Glasgow Biomedical Research Centre, University of Glasgow120 University Place, Glasgow G12 8TA, UK; 3 WPI, Immunology Frontier Research Center, Osaka University3-1 Yamadaoka, Suita, 565-0871, Osaka, Japan

## Abstract

Aurora kinases compose a family of conserved Ser/Thr protein kinases playing essential roles in eukaryotic cell division. To date, Aurora homologues remain uncharacterized in the protozoan phylum Apicomplexa. In malaria parasites, the characterization of Aurora kinases may help understand the cell cycle control during erythrocytic schizogony where asynchronous nuclear divisions occur. In this study, we revisited the kinome of *Plasmodium falciparum* and identified three Aurora-related kinases, Pfark-1, -2, -3. Among these, Pfark-1 is highly conserved in malaria parasites and also appears to be conserved across Apicomplexa. By tagging the endogenous *Pfark-1* gene with the green fluorescent protein (GFP) in live parasites, we show that the Pfark-1–GFP protein forms paired dots associated with only a subset of nuclei within individual schizonts. Immunofluorescence analysis using an anti-α-tubulin antibody strongly suggests a recruitment of Pfark-1 at duplicated spindle pole bodies at the entry of the M phase of the cell cycle. Unsuccessful attempts at disrupting the *Pfark-1* gene with a knockout construct further indicate that Pfark-1 is required for parasite growth in red blood cells. Our study provides new insights into the cell cycle control of malaria parasites and reports the importance of Aurora kinases as potential targets for new antimalarials.

## Introduction

Malaria is one of the most important infectious diseases in humans and represents a major health and economic problem in developing countries. It is caused by infection with obligate intracellular parasites of the genus *Plasmodium.* The *Plasmodium falciparum* species is responsible for most lethal cases of human malaria and the emergence and spread of resistance against the available antimalarial armamentarium creates an urgent need for the development of new treatments (Ridley, 2002). The life cycle of malaria parasites alternates developmental stages with intense cell division and stages where the cell cycle is arrested, implying the existence of an efficient cell cycle control machinery. How *Plasmodium* regulates cell growth and division remains, however, largely unknown. Malaria parasites are at variance from classical mitosis in that they undergo successive nuclear divisions in the absence of cell division to form a multinucleated schizont (reviewed in Bannister *et al*., 2000). The nuclear membrane of malaria parasites is maintained throughout the cell division process, a feature shared by other unicellular eukaryotes such as yeast. Another peculiarity of malaria parasites is that nuclei in a given schizont appear to divide asynchronously as evidenced by the Gaussian distribution of nuclear body numbers in schizonts (whereas synchronous nuclear division would result in a distribution where schizonts with 1, 2, 4, 8 or 2*^n^* nuclear bodies would be prominent) and by the presence of mitotic spindles at various stages of development within a single schizont (Read *et al*., 1993). A clear correspondence between the G1, S, G2 and M phase of the cell cycle has not been established, and the organization of the *Plasmodium* cell cycle during erythrocytic schizogony is as yet not understood. Important divergences in the composition and properties of the cell cycle machinery such as cyclin-dependent protein kinases (CDKs) and cyclins have also been reported (Leete and Rubin, 1996; Doerig *et al*., 2002). To date, few experimental data are available that assign clear roles for the *Plasmodium* CDK-related kinases in cell division (Chen *et al*., 2006; Halbert *et al*., 2010).

Eukaryotic cell mitosis is organized around a microtubule (MT)-organizing centre, typically a centrosome containing two centrioles and their surrounding pericentriolar material, the site of MT assembly. During S phase the centrosome duplicates concomitantly with DNA replication. When mitosis begins, the duplicated centrosomes separate and undergo a process called maturation that consists of the recruitment of proteins involved in MT nucleation from α/β-tubulin dimers. An array of MTs is thus generated and will form the bipolar mitotic spindle after migration of the centrosomes to opposite sides of the nucleus. After nuclear envelope breakdown, in some eukaryotes the condensed chromosomes bind to the spindle MTs at their kinetochores, the sister chromatids split and move to either pole. The chromosomes then decondense, new nuclei are formed, the mitotic spindle is disassembled and cytokinesis is initiated to produce two daughter cells. The spindle pole body (SPB), a trilaminar plaque embedded in the nuclear envelope, is the centrosome equivalent in fungi and malaria parasites (Moore and Sinden, 1974; Schrevel *et al*., 1977; Jaspersen and Winey, 2004). Execution of these mitotic events involves the concerted action of several conserved Ser/Thr protein kinases known as mitotic kinases (Nigg, 2001). In addition to CDKs, polo-like kinase and Nima-related kinase families, mitotic kinases include the Aurora-related kinases, the functions of which are closely related to bipolar MT spindle dynamics (Carmena and Earnshaw, 2003). Investigation of this important mitotic kinase family in malaria parasites may provide clues to *Plasmodium* cell cycle regulation.

Homologues of Aurora-related kinases have been reported in various organisms (Carmena and Earnshaw, 2003). Metazoans have two distinct Aurora family members, Aurora A and B, which are expressed in all cell types where they regulate cell cycle progression from G2 to cytokinesis. A third member, Aurora C, has arisen from Aurora B through gene duplication during mammalian evolution and is specifically expressed in the testis. Aurora A, or the polar Aurora, was initially identified in *Drosophila*. Mutation of the Aurora A gene in *Drosophila* results in the formation of monopolar rather than bipolar mitotic spindles (Glover *et al*., 1995). Consistent with a role in the regulation of spindle formation, Aurora A associates with the centrosome during S phase and becomes heavily concentrated at the spindle poles and detectable along the spindle MTs during mitosis (Marumoto *et al*., 2005). Aurora B, the equatorial Aurora, is required for chromosome condensation, chromosome segregation and cytokinesis. Aurora B displays a ‘chromosomal passenger’ localization pattern: it is associated with kinetochores from prophase to metaphase, and then translocates to the central-spindle MT at anaphase (Vader *et al*., 2006). The yeast *Saccharomyces cerevisiae* and *Saccharomyces pombe* have a single Aurora family member, *Ipl1* (Increases in ploidy) and *Ark1*, respectively, which have localization and functions in yeast cells similar to Aurora B in metazoans (Francisco and Chan, 1994; Leverson *et al*., 2002). So far, no Aurora kinases have been characterized in the early divergent protozoan phylum Apicomplexa, which in addition to the haemosporin *Plasmodium* includes a large number of medically and veterinary important parasites such as the piroplasms *Theileria* and *Babesia*, and the eimerids *Toxoplasma* and *Cryptosporidium*.

In this study we revisited the *P. falciparum* kinome and identified three *P. falciparum* Aurora-related kinases. One of these, *Pfark-1*, is highly conserved in human and rodent malaria species and appears to have a conserved orthologue in apicomplexan parasites whose genome sequence is available. We modified the endogenous *Pfark-1* gene of the 3D7 *P. falciparum* parasite by fusing it to the green fluorescent protein (GFP) and found that the GFP–Pfark-1 protein has an apparent transient localization at duplicated SPBs of only a subset of nuclear bodies initiating bipolar mitotic spindle formation during erythrocytic schizogony. Attempts to produce *P. falciparum* parasites with a disrupted *Pfark-1* gene by homologous recombination were unsuccessful, strongly suggesting that the gene is essential. Given the conserved functions of Aurora kinases in eukaryotes, altogether our data identify Pfark-1 as an A-type Aurora kinase required at the S to M phase transition of the cell cycle.

## Results

### Identification of three Aurora-related kinases in *P. falciparum*

**Fig. 1:**
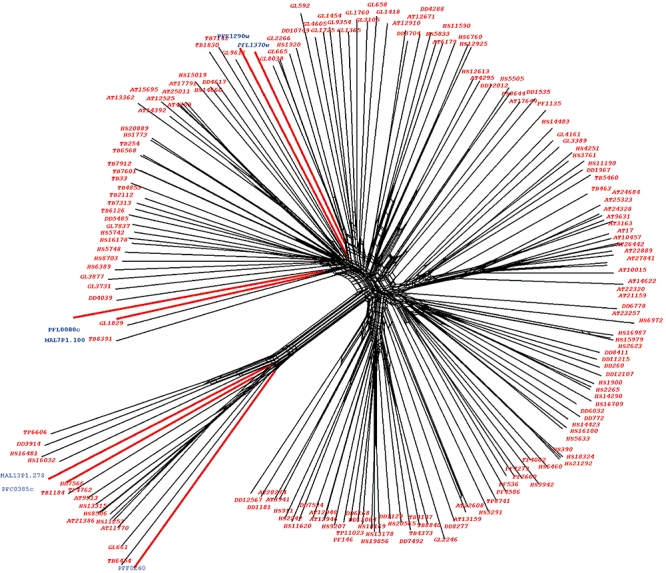
Phylogenetic analysis of *P. falciparum* Aurora kinases. Neighbour-Net tree (Splits-Tree; Huson and Bryant, 2006) of Aurora kinases detected in model genomes. *P. falciparum* sequences appearing in the tree are labelled by capital letters. *P. falciparum* Aurora and Nima-related sequences are indicated in blue and red colour branch. A cluster of sequences containing three *P. falciparum* sequences (PFF0260w, PFC0385c and MAL13P1.278) is clearly separated from the other sequences within the tree. Annotations associated with sequences from this branch indicate that this cluster consists of exclusively of Aurora kinases and a small set of four Polo kinases from Human (HS16481, HS16032), *Dictyostelium discoideum* (DD3914) and *Thalassiosira pseudonana* (TP6606). The kinase sequences with their UniProt identifier are listed in Table S1. We recommend viewing this file online using a graphics programme enabling magnification, such as Microsoft Office Picture Manager.

In a previous phylogenetic analysis of the kinome of *P. falciparum* (Ward *et al*., 2004), we identified a cluster of four Nima-related protein kinases, with which a fifth protein kinase (PlasmoDB gene ID MAL6P1.56, located on chromosome 6 and now named PFF0260w) was weakly and inconsistently associated. In the *P. falciparum* kinome analysis by Anamika *et al*. (2005), MAL6P1.56 did not cluster with the Nima family. We performed blastp analysis of the UniProt database using PFF0260w as a query, which yielded members of the Aurora family as top scores. In order to further investigate the association of PFF0260w with the Aurora kinase family and to identify all *P. falciparum* Aurora-related kinases, the catalytic domain of PFF0260w and the *P. falciparum* NIMA kinases were used as queries in blastp searches of the genomes of *P. falciparum* and a canonical set of eukaryotic genomes. This allowed the identification of 13 *P. falciparum* kinases related to NIMA or Aurora kinases. The catalytic domains identified in *P. falciparum*, together with those from the other organisms, were subjected to multiple sequence alignment and Neighbour-Net phylogenetic tree construction (Fig. 1). A distinct cluster within the tree, clearly separated from the other kinases, comprises a number of established Aurora-related kinases, including human Aurora A, B and C. This group includes PFF0260w and two additional *P. falciparum* sequences, PlasmoDB gene ID PFC0385c and MAL13P1.278, located on chromosome 3 and 13 respectively. None of the four *P. falciparum* Nima-related kinases is included in the ‘Aurora clade’, supporting the contention that PFF0260w is distant from the Nima kinases. Aurora kinases assignments in *P. falciparum* were confirmed by a combination of an *ad hoc* Aurora kinase domain-specific HMM and complete linkage clustering with characterized Aurora kinase catalytic domains from higher eukaryotes.

**Fig. 2:**
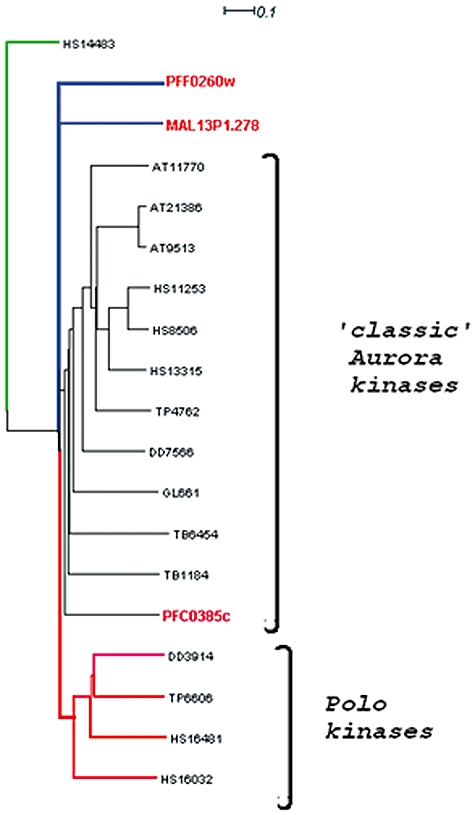
Phylogenetic tree of Aurora-related kinases from the canonical species. The core kinase domains of sequences located within the ‘Aurora clade’ identified above plus an outgroup kinase domain from HS14483 (Uniprot Q9NRH2) were subjected to optimized multiple sequence alignment as described in *Experimental procedures*. A rooted neighbour joining phylogram was generated (Huson and Bryant, 2006). Kinase domains associated with Polo-box duplicated regions (Pfam profile PF00659) and those apparently forming part of ‘classic’ Aurora kinases are indicated.

The sequences associated with the ‘Aurora’ clade along with an outgroup sequence (HS14483, Uniprot Q9NRH2, SNRK_HUMAN SNF-related serine/threonine-protein kinase) were aligned and a rooted neighbour joining phylogram generated (Fig. 2). The kinase domains of PFF0260w and MAL13P1.278 are excluded from the clade containing the ‘classic’ Aurora kinase domains, suggesting they may have diverged from Aurora kinases at a relatively early stage of Aurora kinase evolution. It is of interest that a distinct subcluster appears within the ‘Aurora clade’, consisting of kinase domains from Human, *Dictyostelium discoideum* and *Thalassiosira pseudonana*, in which the Human and *Dictyostelium* proteins are annotated as Polo type kinases. Pfam analysis (Finn *et al*., 2006) of the *Thalassiosira* sequence confirms the presence of the Polo-box duplicated region, diagnostic of Polo type kinases. There is evidence that *Arabidopsis thaliana* and *P. falciparum* lack polo type kinases (Van Damme *et al*., 2004; Ward *et al*., 2004; Hodges *et al*., 2010) consistent with this observation. In contrast, *Trypanosoma brucei* (Uniprot Q15882, Q57VI0) and *Giardia lamblia* (Uniprot A8BPK8) have kinases with Polo-box duplicated regions combined with kinase domains that do not cluster with the set above (data derived from Pfam http://pfam.sanger.ac.uk//family/PF00659#tabview=tab6).

**Fig. 3:**
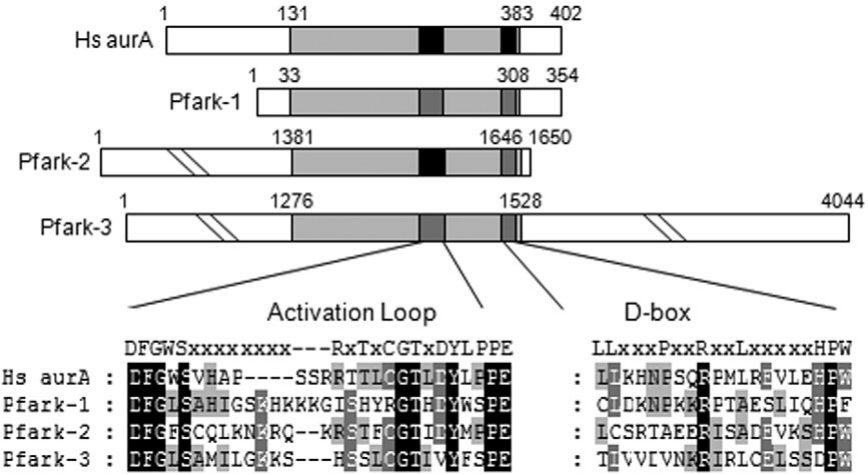
Schematic representation of *P. falciparum* Aurora-related kinases and sequence alignment of conserved motifs of Aurora kinases. Large grey box, catalytic domain; small black (conserved) and grey (not conserved) boxes, activation loop and destruction box. Alignment of Pfark-1 (PFF0260W), Pfark-2 (PFC0385c) and Pfark-3 (MAL13P1.278) structural features as compared with that of human Aurora A (Hs aurA) kinase.

PFF0260w (Pfark-1) is a 40.9 kDa predicted protein and shares the common structure of Aurora-related kinases comprising a catalytic domain and short N-terminal and C-terminal domains (Fig. 3). PFC0385c (Pfark-2) and MAL13P1.278 (Pfark-3) are larger proteins of 193.8 kDa and 475.7 kDa with long N-terminal or N- and C-terminal extensions respectively. Pfark-2, the *P. falciparum* Aurora-related kinase clustering with ‘classic’ Aurora kinases, contains a sequence very similar to the conserved motif found in the potential activation loop between subdomains VII and VIII, DFGWSxxxxxxxxRxTxCGTxDYLPPE, considered as a signature for Aurora kinases. This motif is found neither in Pfark-1 nor in Pfark-3. All three *P. falciparum* Aurora-related kinases have an only partially conserved C-terminal destruction box (D-box) found in all members of the Aurora kinase family.

### Pfark-1 is conserved in apicomplexan parasites

**Fig. 4:**
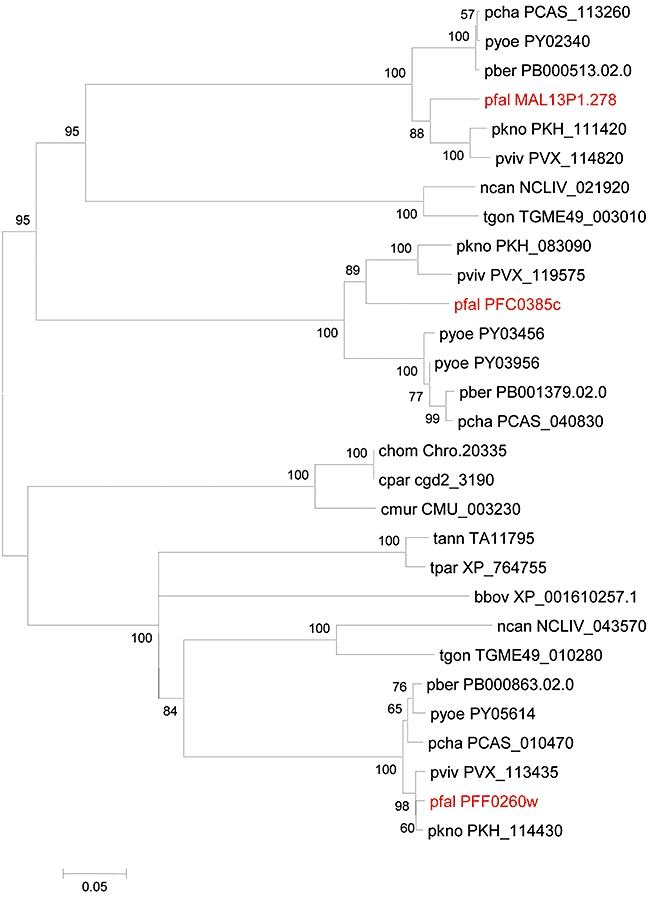
Phylogenetic tree of Aurora-related kinases from Apicomplexa. Phylogram (mega; Tamura *et al*., 2007) with significant bootstrap values (100 iterations). The sequences are labelled with their identifier in the ApiDB database. The core kinase domains of Aurora sequences from the indicated species were extracted and subjected to optimized multiple sequence alignment as described in *Experimental procedures*. Organism groups are Haemosporin (*P. falciparum*, *P. knowlesi*, *P. vivax*, *P. berghei*, *P. yoelii*, *P. chabaudi*), Piroplasm (*T. parva*, *T. annulata*, *B. bovis*) and Coccidia (*T. gondii*, *N. canina*, *C. hominis*, *C. parvum*, *C. muris*).

To explore the conservation of Aurora-related kinases in malaria parasites and other Apicomplexa, we performed an alignment of Pfark-1, -2, -3, and their closest homologues in other available apicomplexan predicted proteomes, and constructed a phylogenetic tree (Fig. 4). According to this analysis, orthologues are present both in human and rodent *Plasmodium* species, having 96–98%, 81–85% and 89–91% amino acid sequence identities over the catalytic domain to Pfark-1, -2 and -3, respectively, indicating that all three Aurora-related kinases are highly conserved in malaria parasites. In addition, except for the Pfark-2 orthologue of *Plasmodium vivax* which has a short predicted N-terminal extension, the overall structures with variable N- and C-terminal extensions are conserved, strongly suggesting conserved functions of Aurora kinases in *Plasmodium* spp. *Babesia*, *Theileria* and *Cryptosporidium* were found to contain a single gene encoding Aurora-related kinases with short N- and C-terminal extensions. The single Aurora in *Babesia* and *Theileria* appears to be a probable Pfark-1 orthologue whereas that of *Cryptosporidium* is either unique to this species or a distant Pfark-1 orthologue. *Toxoplasma* and *Neospora* contain two genes encoding proteins structurally and phylogenetically related to Pfark-1 and Pfark-3. The level of conservation of Pfark-1 in *Toxoplasma*, 62% identity, suggests similar functions in this medically important pathogen. It is notable that the conserved phosphorylatable residue within the T-loop of all members of the Aurora family is not conserved in Pfark-1 and orthologues from other Apicomplexa (Table S2). Instead, Ser/Thr residues present at positions corresponding to residue 198 and 287 of human Aurora A are highly conserved among Apicomplexa. Interestingly, Pfark-2 seems to be unique to *Plasmodium* spp. A recent *Pfark-2* gene duplication event occurred in the rodent malaria species *Plasmodium yoelii*, leading to two genes in tandem, one of which, Py03456, has a truncated N-terminal extension without an in-frame initiation codon and is likely to be a pseudogene. In view of the relative conservation of Pfark-1 across Apicomplexa, we decided to investigate its function using a reverse genetics approach.

### Pfark-1 displays the polar localization of an Aurora A kinase

**Fig. 5:**
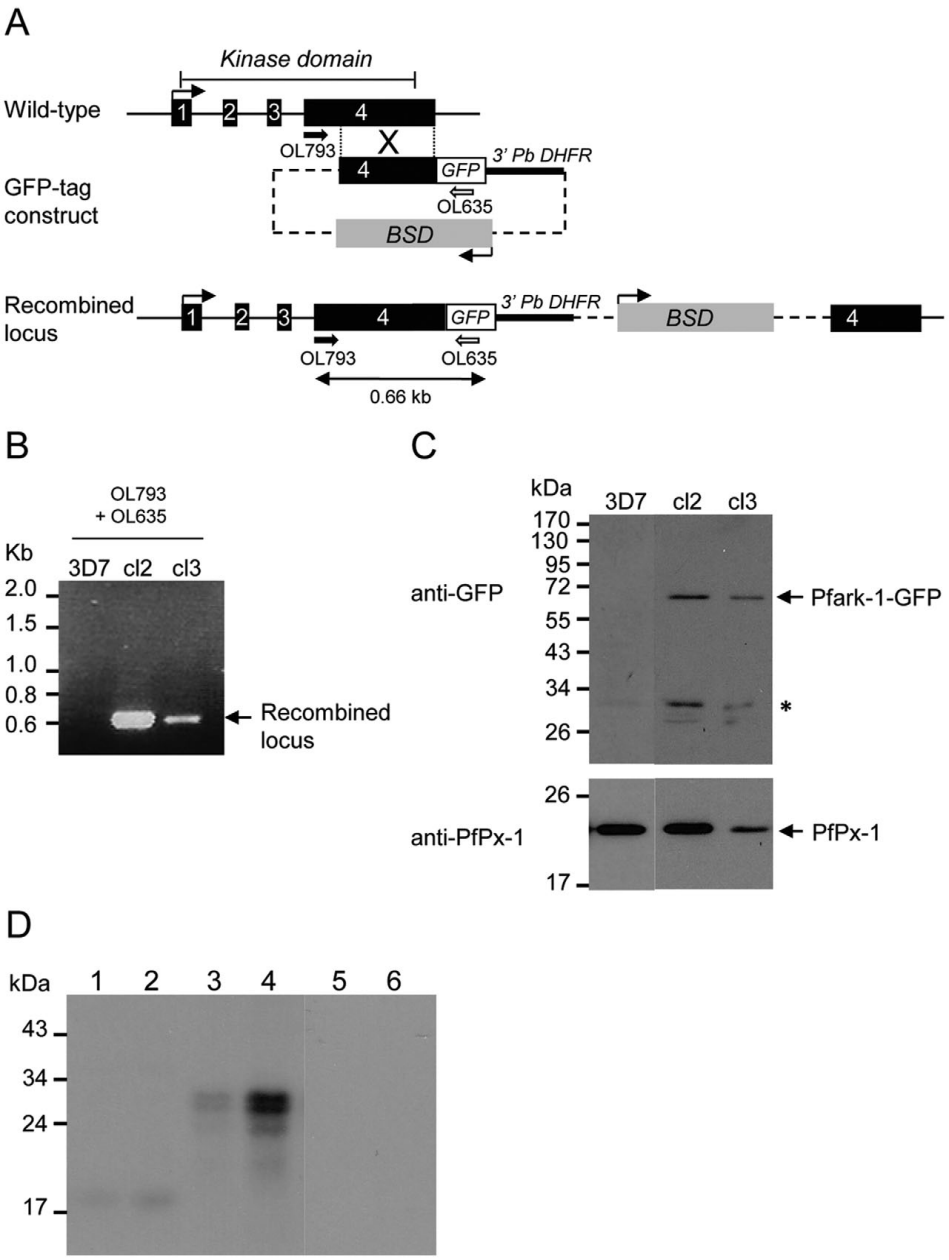
GFP-tagging of *Pfark-1*. A. Schematic representation of the single-cross-over homologous recombination strategy, showing the wild-type *Pfark-1* locus, the GFP-tag construct containing ∼500 bp of the 3′ end coding sequence of *Pfark-1* fused in-frame with the GFP sequence, and the recombined *Pfark-1* locus resulting from integration of the GFP-tag construct. Transcription termination and polyadenylation of the *Pfark-1–GFP* transgene are ensured by the presence of the *P. berghei* dihydrofolate reductase 3′ UTR (3′ Pb DHFR). The location of primers used for PCR analysis, OL793 and OL635, is indicated. BSD, blasticidin-resistance cassette. B. Diagnostic PCR for homologous recombination. Amplification of a 0.66 kb PCR product using OL793 and OL635 as primers and genomic DNA prepared from parasite clones 2 and 3 (cl2 and cl3) indicates integration of the GFP-tag construct in the *Pfark-1* locus. 3D7, wild-type *P. falciparum* parasite. C. Western blot of wild-type 3D7 and Pfark-1–GFP transgenic clones 2 and 3 (cl2 and cl3), using mouse anti-GFP mAbs (Roche). The bottom panel shows an image of the same blot stripped and reprobed with rabbit anti-*P. falciparum* 2 Cys-peroxyredoxin (PfPx-1) antibodies, used as a loading control. Approximately 10 µg of protein extracts from synchronized schizonts were loaded per lane. The predicted molecular weight of the Pfark-1–GFP protein is 70 kDa. The position of Pfark-1–GFP and PfPx-1 is indicated. A ∼30 kDa Pfark-1–GFP degradation product is marked with an asterisk. D. SDS-PAGE autoradiography of kinase assays of anti-GFP (Chromotek) immunoprecipitates from 3D7 wild-type control (lane 1, 3) or Pfark-1–GFP transfectants (lane 2, 4), using mixtures of MBP and histone H1 (1, 2, 5) and α- and β-casein (lane 3, 4, 6) as substrates. Kinase reaction in absence of immunoprecipitates (substrate alone) provides another negative control (lane 5, 6).

Transgenic *P. falciparum* parasites expressing a C-terminally GFP-tagged Pfark-1 protein were generated using a single-cross-over homologous recombination strategy (Fig. 5A). The gene encoding Pfark-1 was targeted using a GFP-tag construct based on the *P. falciparum* transfection plasmid pCam-BSD (Mamoun *et al*., 1999). Blasticidin-resistant parasite populations were obtained within 3 weeks after transfection. After successful integration of the GFP-tag construct into the *Pfark-1* locus was ascertained by PCR analysis, the transfectants were cloned by limiting dilution and maintained in the presence of blasticidin. Diagnostic PCR for integration of the GFP-tag construct in clones 2 and 3 is shown in Fig. 5B. These two clones were used for further analyses. Western blot analysis using anti-GFP mAbs confirmed the in-frame fusion of the GFP-tag to Pfark-1 (Fig. 5C). A ∼70 kDa product corresponding to the Pfark-1–GFP (expected size 68.9 kDa) was detected in schizont extracts prepared from transgenic parasites but not wild-type 3D7 parasites. An additional anti-GFP reactive species of ∼30 kDa was also observed and is likely a degradation product of Pfark-1–GFP. None of the two clones exhibited any growth defect as compared with the wild-type 3D7 parasite (data not shown), indicating that the addition of a C-terminal GFP-tag did not affect asexual multiplication of the parasite in erythrocytes. Enzymatic activity associated with the Pfark-1–GFP fusion protein was assessed after immunoprecipitation using anti-GFP beads. As shown Fig. 5D, detection of a high level of α- and β-casein (known substrates of Aurora family members) kinase activity in immunoprecipitates from Pfark-1–GFP transfectants as compared with that obtained with 3D7 control parasites suggests that the Pfark-1–GFP fusion protein is active *in vivo*.

**Fig. 6:**
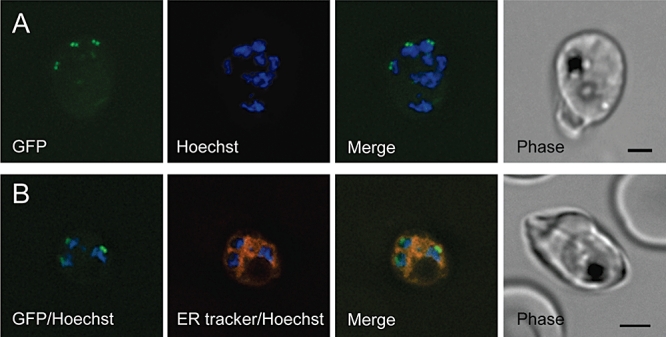
Subcellular localization of Pfark-1–GFP. Live images of schizont stage *P. falciparum* transgenic parasites expressing the Pfark-1–GFP protein. A. Parasites stained with Hoechst 33258. The localization of C-terminally GFP-tagged Pfark-1 protein (green signal) was observed as paired dots in close proximity to Hoechst-stained nuclear DNA (blue). B. Parasites stained with Hoechst 33258 and ER-tracker. The ER forms a highly developed membranous system from trophozoite to schizont stage. The superimposed images show that the GFP-tagged Pfark-1 protein (green dots) localize to the edge of the nuclear DNA (blue) and the ER/nuclear membrane (red), suggesting a localization of Pfark-1–GFP in contact with the nuclear envelope. Identical results were obtained with clone 2 and clone 3. All images were acquired using a Deltavision RT wide-field epifluorescence microscope imaging system and a 100×/1.4 objective and processed using SoftWoRx deconvolution software. The image analysis software used was imaris version 5.0. Corresponding phase-contrast images are shown as well.Scale bars for (A) and (B), 2.0 and 2.5 µm respectively.

Fluorescence microscopy of live transgenic parasites consistently provided images of schizonts with paired dots of concentrated Pfark-1–GFP protein associated with a subset of Hoechst-positive nuclei (Fig. 6A). No punctuate GFP fluorescence was observed in ring and trophozoite stages. The paired dots appear in juxtaposition to the nuclei and suggest an association with SPBs embedded in the nuclear membrane. To analyse the nuclear localization in more detail, the parasite endoplasmic reticulum (ER) was stained with a fluorescent dye, ER-Tracker™ Red, which provides specificity for ER membranes. Figure 6B shows an image where two Pfark-1–GFP spots are in the same focal plane as the ER staining and appear to localize inside the space delineated by the ER/nuclear membrane and the Hoechst-stained nuclear DNA, suggesting a close contact to the nuclear envelope and a localization at the nuclear side of the nuclear membrane.

### Pfark-1 identifies nuclei undergoing early mitotic spindle formation

**Fig. 7:**
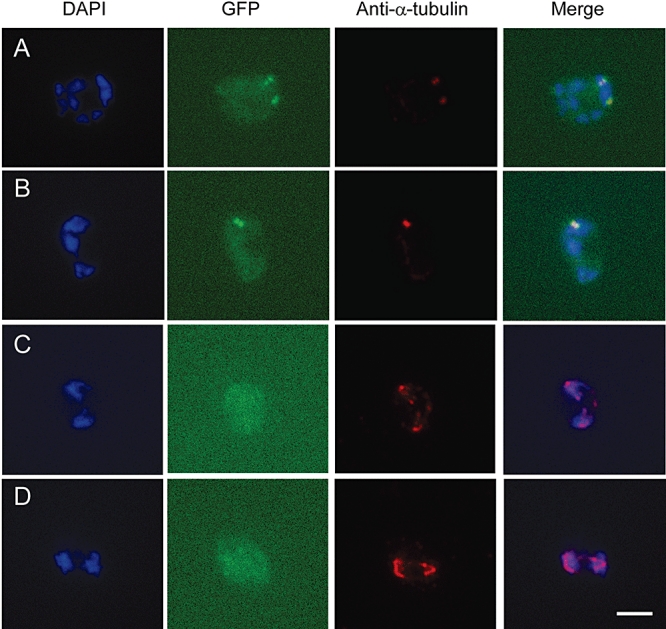
Colocalization experiments of Pfark-1–GFP with α-tubulin. Fluorescence microscopy of fixed schizont stage transgenic parasites expressing Pfark-1–GFP. A and B. An anti-α-tubulin mAb (DM1A) recognizes discrete foci (red) near DAPI-stained nuclear DNA (blue). The red fluorescent pattern is consistent with early-stage mitotic spindle formation developing from the spindle pole bodies anchored in the nuclear membrane. In colour merge images, the Pfark-1–GFP protein appears to localize at opposite sides of the early mitotic spindle, consistent with a localization at the mitotic spindle poles. C and D. In these two panels the anti-α-tubulin mAb DM1A (Sigma) recognizes partly disassembled bipolar mitotic spindles (red) aligned with DAPI-stained nuclear bodies (blue), representing daughter nuclear bodies in the process of completion of nuclear division. The Pfark-1–GFP protein does not appear now to be confined to mitotic spindle poles. A diffuse cytosolic green fluorescence is observable, presumably representing the GFP degradation product observed by Western blot analysis. All images were acquired, processed and analysed as in legend to Fig. 6. Scale bar, 2.5 µm.

To determine whether the nuclear envelope structures with which Pfark-1 associates are SPBs, the Pfark-1–GFP transgenic parasites were stained with the mouse anti-chicken α-tubulin mAb clone DM1A (Sigma), which is known to cross-react with *P. falciparum*α-tubulin (Fowler *et al*., 2001; Singh *et al*., 2007). The use of anti α-tubulin antibodies in immunofluorescence allowed Read *et al*. (1993) to monitor the development of mitotic spindle MTs, from the dot-like spindle centriolar plaque (or SPB) to the full bipolar mitotic spindle. As shown in Fig. 7A and B, the anti-α-tubulin antibody reacts with dotted paired or elongated single MT structures associated with a subset of nuclei, which in the superimposed image appear to be precisely those nuclei that are associated with the Pfark-1–GFP protein as well. The localization of Pfark-1–GFP at opposite sides of these small mitotic MT structures strongly suggests Pfark-1 recruitment at duplicated SPBs at an early stage of bipolar MT spindle formation. In marked contrast, no punctuate GFP staining of the spindle poles was detectable at later stages of nuclear division cycle, when fully developed spindles are visible (Fig. 7C and D).

**Fig. 8:**
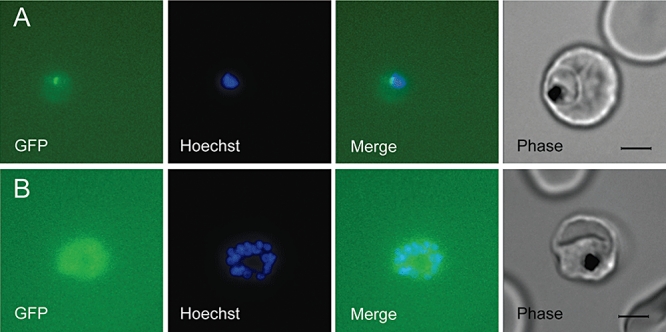
Pfark-1–GFP expression in early and late schizogony. A. Live images of Pfark-1–GFP transgenic parasite undergoing the first mitotic division of schizogony. A single Hoechst-stained nuclear body (blue) is associated with two discrete paired dots (green) representing the Pfark-1–GFP protein. B. Live images of post-mitotic Pfark-1–GFP transgenic parasite. In late schizogony, the parasites have completed their mitotic divisions and are in a differentiation phase leading to the formation of new merozoites. At this stage, the Hoechst-stained nuclear bodies (blue) become much more sharply defined. No concentrated dotted Pfark-1–GFP fluorescence is detectable. All images were acquired, processed and analysed as in legend to Fig. 6. Scale bars, 2.5 µm.

The results from the immunofluorescence analysis strongly suggest that Pfark-1 transiently associates with duplicated SPBs from nuclei at the onset of nuclear mitosis and therefore, point to functions at the mitotic spindle poles such as SPB separation, SPB maturation or the formation of mitotic spindle MTs. To confirm that Pfark-1 associates with nuclear bodies undergoing mitosis, we assessed the expression of the Pfark-1–GFP protein in early schizonts as compared with late schizonts using parasites from highly synchronized cultures. The development of parasites through ring, trophozoite and schizont stages was assessed by Giemsa staining of thin blood smears of parasite cultures (data not shown). As shown in Fig. 8A, concentrated Pfark-1–GFP fluorescence in paired dots was readily detected in mature trophozoites with a single nuclear body, but not in ring or early trophozoites. Given that the Pfark-1–GFP protein appears to concentrate at duplicated SPBs of nuclei developing mitotic spindle MTs, these nuclei are likely to undergo the first mitotic division. In contrast, Pfark-1–GFP spots were not observed in late segmental stages which have terminated their nuclear division and are in the process of merozoite formation (Fig. 8B).

### Asynchrony of nuclear division in *P. falciparum* schizogony

**Fig. 9:**
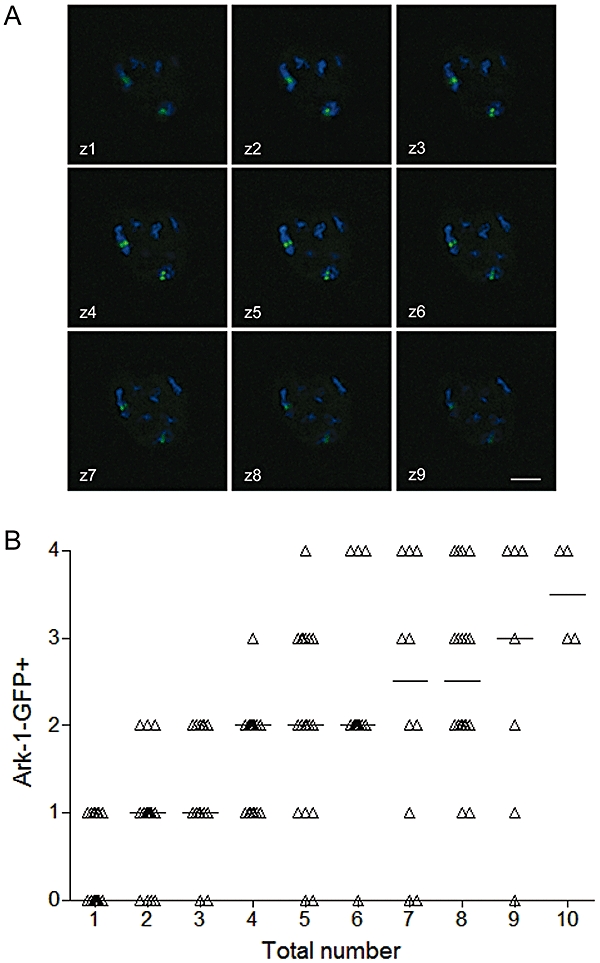
Asynchrony in *P. falciparum* schizogony. A. *Z*-stack live images of a schizont-stage *P. falciparum* transgenic parasite expressing Pfark-1–GFP. Images z1 to z9 represent 0.2 µm *z*-stacks of a single multinucleate schizont showing that the paired dots of Pfark-1–GFP fluorescence (green) are restricted to two out of seven Hoechst-stained nuclear bodies (blue). Scale bar, 2.5 µm. B. Distribution of Pfark-1–GFP positive (Ark-1–GFP^+^) nuclear bodies plotted against total number of nuclear bodies within single parasites undergoing schizogony. Bars indicate median of Ark-1–GFP^+^ nuclear bodies per schizont containing one or up to 10 nuclear bodies. This analysis supports and extends the tubulin immunofluorescence showing asynchrony of nuclear multiplication in a single *P. falciparum* parasite undergoing schizogony. The average occurrence of one Ark-1–GFP^+^ nuclear body in parasites with two nuclear bodies indicates that asynchrony is occurring already at an early stage.All images were acquired, processed and analysed as in legend to Fig. 6.

Our fluorescence microscopy analyses support the asynchrony of nuclear division in parasites undergoing schizogony that was previously suggested by the observation of spindles at different phases of division and a Gaussian distribution of nuclear bodies in a single schizont (Read *et al*., 1993). As a given example (Fig. 9A), *z*-stack images of a single Pfark-1–GFP transgenic schizont clearly reveal two Pfark-1–GFP positive nuclei out of a total of seven nuclei. This staining allowed us to assess the succession of nuclear divisions by plotting the number of GFP-positive nuclei against the total number of nuclei in a single schizont (Fig. 9B). In schizonts with a low number of nuclei, i.e. one to four nuclei, an average of 50% of nuclei are GFP-positive. The relatively broad distribution of GFP-positive nuclei per schizont favours a model of asynchronous mitotic nuclear division rather than reflecting a proportion of nuclei arrested in the cell cycle and unable to participate in subsequent rounds of division. Noteworthy, the average occurrence of one Pfark-1–GFP-positive nucleus in parasites with two nuclei indicates that asynchrony occurs at an early stage. In schizonts with higher numbers (5–10) of nuclei, strikingly, a maximum of four Pfark-1–GFP-positive nuclei can be observed, suggesting that four nuclei at most are in early M phase at any given time. Thus, at these stages, nuclear divisions appear to be asynchronous as well; it is unclear whether a number of nuclei are arrested in the cell cycle. Monitoring individual nuclei within a single Pfark-1–GFP transgenic schizont by time-lapse cell imaging will help resolve this issue.

### Pfark-1 is essential for *P. falciparum* blood-stage cycle

The ability to generate Pfark-1–GFP transgenic parasites using a homologous recombination strategy demonstrates that the *Pfark-1* locus is amenable to genetic manipulation. To investigate whether Pfark-1 plays an essential function in erythrocytic stages, we targeted the *Pfark-1* gene with a KO construct lacking sequences coding for residues essential for the catalytic activity. Single-cross-over integration of the KO construct would generate a pseudo-diploid configuration with both truncated *Pfark-1* copies lacking sequences coding for essential regions of the kinase domain (Fig. S1); this strategy has been used to generate a number of kinase knockouts in *P. falciparum* (Doerig *et al*., 2010). Four independent transfections were performed and blasticidin (BSD)-resistant populations were obtained in each case. PCR analyses of the parasite genotypes did not reveal any disruption of the *Pfark-1* locus, even after maintaining the transfectants for more than 6 months in culture (Fig. S1) (in comparison, integration of the GFP-tagging construct was evident 4 weeks post transfection). Instead, the wild-type locus appeared to remain unmodified and the knockout construct remained episomal, indicating that targeting of the *Pfark-1* gene with a KO construct either is lethal or severely impairs growth rate.

## Discussion

Aurora kinases play pivotal roles in the control of cell division. The deregulation of Aurora kinase functions and their contribution to tumorigenesis has led to a major interest in the development of Aurora kinase inhibitors for cancer therapy (Andrews, 2005). Here, we provide structural and functional evidence for the presence of members of this important mitotic kinase family in the kinome of Apicomplexa. The finding that at least one of these, Pfark-1, appears to be essential for *P. falciparum* blood stage cycle, and hence validated as a potential drug target, indicates that such inhibitors might be considered for the development of new antimalarial. In this regard, a high-throughput screening for *P. falciparum* inhibitors using a 2-million compound library has been recently reported (Gamo *et al*., 2010). Very remarkably, a high proportion of protein kinases, including Pfark-1 and Pfark-2, were identified by chemoinformatics as potential targets.

During the erythrocytic schizogony, malaria parasites undergo multiple rounds of DNA replication and asynchronous nuclear division without cytokinesis. Overall our study reveals that mitotic control mechanisms implicate the finely tuned and transient recruitment to SPBs of an Aurora-related kinase, Pfark-1, during *P. falciparum* schizogony. An important finding is that the Pfark-1–GFP protein can be observed in the form of paired dots (we never saw single dots) in juxtaposition of either paired MT spots or short intranuclear MT spindles. This strongly suggests association of Pfark-1 with duplicated and separated SPBs beginning to nucleate the mitotic MT spindle. Cytoplasmic centrioles adjacent to the nuclear membrane SPBs are sometimes present in other Apicomplexa but not in malaria parasites which lack these structures and construct MT spindles using only the SPBs (Morrissette and Sibley, 2002). During yeast nuclear division, a fibrous link connecting the duplicated SPBs, called the inter-SPB bridge, is severed in late S phase. The separated SPBs then move apart until they come face to face and assemble a characteristic short intranuclear spindle (Lim *et al*., 2009). If a similar mechanism operates during *Plasmodium* nuclear division, this would imply that by the time Pfark-1 is recruited at the SPBs, the SPBs are already duplicated and separated. This would imply a recruitment of Pfark-1 at SPBs upon initiation of M phase of the cell cycle. A clear correlation between *Plasmodium* erythrocytic developmental stages and the classical eukaryotic cell cycle phases is not established. It has been suggested that the ring and early trophozoite stages correspond to G1, and the S phase is initiated in late trophozoites. During the erythrocytic schizogony, nuclei are thought to undergo successive rounds of S/M phases without gap phases, since γ-irradiation of schizonts does not result in a reduction of DNA synthesis despite DNA damage. In response to DNA damage, eukaryotic organisms activate G1/S, intra-S and G2/M cell cycle checkpoints. Despite an apparent lack of this checkpoint in malaria parasite, the cores of eukaryotic nucleotide excision and homologous recombination repairs are present, suggesting the availability of DNA repair pathways (Gardner *et al*., 2002). Whether the nuclei are back in G1 in late segmenter is unclear as well. An intriguing observation, provided by the quantitative analysis of nuclei associated with the Pfark-1–GFP protein, is that a maximum of four Pfark-1–GFP-positive nuclei can be observed in individual schizonts. Interestingly, a consistently even number of daughter merozoites in mature segmenters, with an average of 16–20 per parasite, has been reported by Reilly *et al*. (2007). How asynchronous schizogony results in an even number of daughter merozoites might be explained by our present study suggesting that four nuclei are undergoing S/M cycle at a given time. This further suggests that although the nuclear divisions appear asynchronous, strict checkpoint mechanisms may control how many nuclei can re-enter the cell cycle. Monitoring individual nuclei within a single Pfark-1–GFP transgenic schizont by time-lapse cell imaging will be of crucial importance in clarifying this matter.

The defining characteristic of Aurora A subfamily members is their association with centrosomes and regions of MTs that are proximal to the centrosome (Marumoto *et al*., 2005). Given its MT spindle pole localization, Pfark-1 might therefore be a functional homologue of metazoan Aurora A. The emerging picture of centrosomes and their fungal SPB equivalent is that these structures can exert a control over the cell cycle by providing a scaffold for cell cycle regulators (Doxsey *et al*., 2005). During G2-M progression in metazoans, major mitotic regulators, including cyclin B–Cdk1 complex, Polo-like kinase Plk1, as well as Aurora A, accumulate at centrosomes, allowing their co-ordinated and efficient co-activation and promoting entry of cells into mitosis. Our observation that in malaria parasites an essential Aurora-related kinase is recruited at mitotic spindle poles during nuclear divisions is the first evidence supporting a conserved mechanism of cell cycle control by mitotic kinases at the SPB in malaria parasites. In fact, in other organisms mitotic entry is essentially controlled by Cdk1, whose activity is regulated directly by activators such as mitotic cyclins and the phosphatase Cdc25, and indirectly by Plk1 and Aurora A. Of the six *P. falciparum* CDK-related kinases, PfPK5 is that which displays the closest similarity and phylogenetic relatedness to Cdk1 homologues, Cdk1, Cdk2 and Cdk3, of higher eukaryotes (Le Roch *et al*., 2000; Ward *et al*., 2004). The expression pattern of PfPK5, with a peak of mRNA, protein levels and activity in late trophozoites and schizonts (Graeser *et al*., 1996), is compatible with a role in nuclear division cycle and make PfPK5 a good candidate for being a major cell cycle regulator. In human cells, Aurora A is required for the initial recruitment of cyclin B–Cdk1 at the centrosome. Since PfPK5 localizes as discrete spots at the periphery of the DNA in parasites in the process of nuclear division (Graeser *et al*., 1996), it will be of great interest to investigate its localization with respect to that of Pfark-1. Interestingly, in malaria parasites there are no candidates for Polo-like kinases, neither are there strong candidates for the network of proteins regulating the activation of the cyclin B–Cdk1 complex such as the mitotic cyclin-CDK inhibitory kinases Wee1 and Myt1 and the Cdc25 phosphatase (Ward *et al*., 2004; Wilkes and Doerig, 2008). Pfmrk, a putative cyclin-CDK activator kinase (CAK), appears not to possess CAK activity towards PfPK5 (Le Roch *et al*., 2000; Chen *et al*., 2006). This is consistent with observations that PfPK5 regulation differs in important aspects from that of homologues in human and yeast (Le Roch *et al*., 2000). However, the Thr and Tyr residues that are the targets of Wee1 and Myt1 whose phosphorylation inactivates Cdk1 in other eukaryotes are conserved in PfPK5 and other *P. falciparum* Cdk-related kinases, suggesting the possibility that unidentified functional homologues of these regulators may be present in malaria parasites. Another key player in mitosis entry is the *Aspergillus* Nima/mammalian Nek-2, which is required for centrosome separation at G2/M transition, and in *Aspergillus* also controls nuclear pore complex disassembly so that tubulin can get access to the nucleus to assemble the mitotic spindle (in fungi, like in Apicomplexa, the nuclear membrane does not break down during mitosis). We have previously shown that the *P. falciparum* kinome includes four Nima-related kinases; one of these, Pfnek-1, is expressed in asexual stages and clusters within the *Aspergillus* Nima/human Nek-2 branch in phylogenetic trees (Reininger *et al*., 2005). The *Toxoplasma gondii* orthologue TgNek-1 seems to exhibit spindle pole association during mitosis (Gubbels *et al*., 2008a), supporting partial conservation of regulatory mechanisms for mitotic entry in Apicomplexa and other eukaryotes. In agreement with this conclusion, a forward genetic approach in *Toxoplasma* identified a TgNek1 mutation causing severe mitotic defects as evidenced by disorganized mitotic spindle apparatus and DNA over-replication (Gubbels *et al*., 2008b).

After they duplicate and separate, centrosomes (and most likely *Plasmodium* SPBs) must recruit a number of different proteins in a process known as maturation. This process occurs at the G2/M transition and promotes mitotic spindle assembly. In the absence of Aurora A, the recruitment to the centrosome of several components of the pericentriolar matrix (PCM), including γ-tubulin, is deficient. It will be therefore of great interest to determine whether the recruitment of the Pfark-1–GFP protein at SPBs during the schizogony coincides with an accumulation of γ-tubulin at these sites. The recruitment of Pfark-1 at duplicated SPBs also raises the questions of how Pfark-1 is targeted to the SPBs, and whether and how is it activated. The targeting of Aurora A to mammalian centrosomes requires the Polo-like kinase 1 (Plk1), which is also implicated in centrosome maturation and in the control of mitotic entry (Archambault and Glover, 2009). Aurora A and a Plk1-interacting protein, Bora, have been shown to cooperatively activate Plk1, hence Plk1 and Aurora A are forming a positive feedback loop for mitotic entry (Seki *et al*., 2008). A direct Aurora A regulator at the centrosome is the protein phosphatase type 1 (PP1), which forms a complex with Aurora A and inhibits its kinase activity (Katayama *et al*., 2001). *P. falciparum* contains a PP1 protein phosphatase with high sequence similarity to PP1 members in other species (Wilkes and Doerig, 2008). Studies in other eukaryotes have demonstrated the ability of Aurora A substrates to contribute to the localization and activation of Aurora A through phosphorylation-independent mechanisms ensuring spatial and temporal control of the Aurora A kinase activity. The mechanism is best understood for the localization and activation of Aurora A at spindle MTs, both of which have been shown to depend on a substrate of Aurora A, the microtubule-associated protein (MAP) termed Tpx2 (Kufer *et al*., 2002; Eyers *et al*., 2003). A study by Bayliss *et al*. (2003) has revealed how the binding of Tpx2 to Aurora A induces a conformational change of Aurora A. Proteins associating and interacting with Aurora A at the centrosome such as the centrosomal protein centrosomin (Terada *et al*., 2003), the LIM domain-containing protein Ajuba (Hirota *et al*., 2003) and the transforming acidic coiled coil protein TACC (Giet *et al*., 2002), however, are not identifiable on the basis of sequence homology. Future work on Pfark-1 will be to dissect out its substrate specificity and regulation by immmunoprecipitation analyses. Immunoprecipitation from cellular extracts of metazoans has identified multiple Aurora A-binding proteins such as PP1, Ajuba and Tpx2. The identification and characterization of molecules involved in *P. falciparum* centrosome (SPB) function will provide insight into the mechanistic details of parasite multiplication. In this context, four *P. falciparum* centrins have been recently identified and characterized (Mahajan *et al*., 2008), providing a significant progress towards our understanding of the cell cycle machinery in malaria parasites. Furthermore, in an attempt to investigate the biochemical and enzymological properties of Pfark-1, we have expressed Pfark-1 as a GST fusion protein in *Escherichia coli*, and found that the purified recombinant protein, unlike other Aurora family members, is unable to autophosphorylate and is not active *in vitro* (L. Reininger and C. Doerig, unpubl. results). We here report that the conserved Thr residue in the activation loop of Aurora members, critically involved in human Aurora A activation (Ohashi *et al*., 2006), is substituted by a histidine in Pfark-1 and its apicomplexan orthologues (Table S2 and Fig. 3); we cannot exclude that this His residue is a phosphorylation site *in vivo*, as some *P. falciparum* genes (e.g. PFD0685c and PF14-0326) display regions with low-level similarity to the histidine kinase domain (Ward *et al*., 2004). Site-directed mutagenesis of the adjacent Ser residue to an acidic residue did not produce an active GST–Pfark-1 fusion protein (L. Reininger and C. Doerig, unpubl. results). However, the presence of a kinase activity in Pfark-1–GFP immunoprecipitates suggests that the protein is activated *in vivo*. Future work aiming at identifying Pfark-1 substrates/regulators may make the Pfark-1 protein kinase amenable to high-throughput screening of chemical libraries.

In conclusion, we have provided phylogenetic and functional evidence for the presence of Aurora kinase homologues in Apicomplexa. Whether Pfark-2 and Pfark-3 are cell cycle stage-dependent and function in mitotic spindle dynamics remains to be determined. Evolutionary analysis of all known Aurora kinases favours a lineage-specific expansion of Aurora kinases in metazoans and plants (Brown *et al*., 2004). Likewise, our present phylogenetic analysis favours a model of independent Aurora kinase gene duplication in Apicomplexa. The different number of Aurora kinases found in Apicomplexa may be related to the various modes of division, malaria parasites undergoing schizogony having an apparent higher level of complexity than parasites undergoing binary fission such as *Babesia* and *Theileria*. Clearly, the identification of members of a major mitotic kinase family is an important step towards a better understanding of the molecular control of mitosis and budding in Apicomplexa. Questions remain in explaining how mitotic kinases regulate progression through the cell cycle and how the cell cycle is linked to parasite development.

## Experimental procedures

### Parasite culture and transfections

The 3D7 clone of *P. falciparum*, its F12 subclone and the 3D7 transfectants described in this article were grown in human erythrocytes as described previously, using 0.5% Albumax II (Invitrogen) instead of human serum (Walliker *et al*., 1987). Parasites were synchronized twice using sorbitol according to Lambros and Vanderberg (1979). To obtain enriched preparations of schizont-stage parasites, mature trophozoites were enriched by using the VarioMACS separator and CS MACS columns (Miltenyi Biotec), resuspended in complete medium and returned to culture conditions for 1–10 h prior to harvesting. Development of schizont stages was monitored by Giemsa-stained thin blood smears. For transfections, synchronized ring-stage parasites (3D7 clone) were electroporated with 50–100 µg of plasmid DNA using standard procedures (Sidhu *et al*., 2005). Transformed parasites were selected in presence of 2.5 µg ml^−1^ blasticidin (Calbiochem), then cloned by limiting dilution.

### Bioinformatics

#### Origin of genomic peptide sets

A canonical set of conceptual translations derived from the published genomes of six eukaryotic species were acquired. The species utilized (and source of the protein sequences) were: *Homo sapiens* (Opisthokonts) ftp://ftp.ensembl.org/pub/release-30/human-30.35c/data/fasta/pep/Homo_sapiens.NCBI35.apr.pep.fa.gz; *D. discoideum* (amoebazoa) http://www.dictybase.org/db/cgi-bin/dictyBase/download/download.pl?area=blast_databases&ID=dicty_primary_protein.gz; *A. thaliana* (viridiplantae) ftp://ftp.tigr.org/pub/data/a_thaliana/ath1/SEQUENCES/ATH1.pep.gz; *P. falciparum* (alveolates) http://www.plasmodb.org/common/downloads/release-5.5/; *T. pseudonana* (heterokonts) ftp://ftp.jgi-psf.org/pub/JGI_data/Diatom/thaps1/thaps1Prots.fasta.gz; *T. brucei* (discicristates) ftp://ftp.sanger.ac.uk/pub/databases/T.brucei_sequences/T.brucei_genome_latest; *G. lamblia* (excavates) http://gmod.mbl.edu/perl/site/giardia?page=download_tool&file=orfs_aa&type=orfs&noheader=T. In addition conceptual translation sequence sets were obtained for apicomplexan species as follows: *Plasmodium* species (*P. berghei, P. chabaudi, P. falciparum, P. knowelsi, P. vivax, P. yeoli*) from PlasmoDB http://plasmodb.org/common/downloads/release-6.3/; *Cryptosporidium* species (*C. hominis, C. parvum*) from CryptoDB http://cryptodb.org/common/downloads/release-4.2/; *Theileria* species (*T. annulata*ftp://ftp.sanger.ac.uk/pub/pathogens/T_annulata/TANN.GeneDB.pep) (*T. parvum*, ftp://ftp.ncbi.nih.gov/genomes/Protozoa/Theileria_parva/); *T. gondii* and *Neospora caninum* sequences from ToxoDB http://toxodb.org/common/downloads/release-6.0/NeosporaCaninum/, http://toxodb.org/common/downloads/release-6.0/Tgondii/; *Babesia bovis* from NCBI –ftp://ftp.ncbi.nih.gov/genomes/. These sequence sets were combined as appropriate (a canonical eukaryote set and an apicomplexan set) and blast (Altschul *et al*., 1997) databases created.

#### Identification of homologues, multiple sequence analysis and phylogenetic analysis

*P. falciparum* sequences of interest were aligned under the guidance of the Pfam profile PF00069 (Finn *et al*., 2006) using the hmmalign facility of hmmer (Eddy, 1998). Alignment output in clustalw format was trimmed down to those blocks encompassing match states to the profile and ungapped Fasta formatted sequences extracted from this subset of the alignment (t-coffee seq_reformat option; Notredame *et al*., 2000), effectively extracting the kinase domains. These were submitted as queries to a blastp search (Altschul *et al*., 1997) of the databases above with an expectation threshold of 10^−30^. All conformant sequences were selected from the canonical sequence set and kinase domains extracted. For the apicomplexan sequences a set of reciprocal best hits to the three *P. falciparum* sequences were determined, and kinase domains extracted. Multiple sequence analysis (MSA) was performed by three independent methods: clustalw (Thompson *et al*., 1994), t-coffee (Notredame *et al*., 2000) and hmmalign (Eddy, 1998) guided by the Pfam kinase profile (PF00069). The alignments used the default settings for each method. Alignments were combined under t-coffee, and quality of alignment assessed. Columns displaying low consistency (score < 5) or significant numbers of gaps (> 15%) were removed. Phylogenies were determined either using the appropriate option of SplitsTree version 4 (Huson and Bryant, 2006) or with mega version 4 (Tamura *et al*., 2007).

### Molecular cloning, plasmid constructs

Genomic and cDNA sequence data were accessed via PlasmoDB (http://www.plasmodb.org). Pfark-1 (gene identifier PFF0260w) entire 1062 bp open reading frame was PCR-amplified from a *P. falciparum* cDNA library (kindly provided by A. Craig) using primers (forward-OL7 GGGGGATCCATGAACGATTCAATC and reverse-OL6 GGGGGTCGACTCATTTGTTGTGAAACCA) designed to contain the start and stop codons as well as BamHI and SalI restriction sites (underlined) respectively. The *Pfark-1* gene fragments of the GFP and KO constructs were amplified from *P. falciparum* genomic DNA (3D7) using primers (forward-OL729 CCCGGGGGTTTATCAGCACATATAGGATC and reverse-OL730 GGATCCTTTGTTGTGAAACCATATGGGTG) designed to contain XmaI and BamHI restriction sites and primers (forward-OL48 GGGGGGATCCGCACATGGTAGTGTATTC and reverse-OL49 GGGGCGGCCGCAGGAGACCAATAATCATG) designed to contain BamHI and NotI restriction sites respectively. The 769 bp Pfark-1-KO fragment was cloned into the pCam-BSD transfection plasmid (Mamoun *et al*., 1999), and the 536 bp Pfark-1–GFP-tag fragment was cloned into a modified pCam-BSD plasmid containing the open reading frame of the *gfp* gene version II subcloned from pHH2 (Waller *et al*., 2000), followed by the 3′ UTR of the *P. berghei dihydrofolate reductase* gene (3′ Pb DHFR) downstream of the multicloning site. All constructs were sent to the Dundee Sequencing Service, University of Dundee, UK, for sequence verification before being used.

### Genotype analysis of 3D7 transfectants

Genomic DNA from transfectants and 3D7 control parasites were extracted from saponin lysis pellets by standard proteinase K digestion in presence of SDS, phenol/chloroform/isoamyl alcohol extraction and ethanol precipitation. Integration of the GFP-tag construct in the *Pfark-1* locus was monitored by a diagnostic PCR using primer pair (forward-OL793 GTCATGATAAAAGAATAGCAC complementary to *Pfark-1* codons 146–153, and reverse-OL635 CAGGTAGTTTTCCAGTAGTGC complementary to codons of the *gfp* coding sequence) amplifying a predicted 0.66 kb PCR product. For analysis of *Pfark-1* KO transfectants, a PCR using the primer pair OL167 (TATTCCTAATCATGTAAATCTTAAA) and OL168 (CAATTAACCCTCACTAAAG) specific for the pCam-BSD vector backbone produced a 0.93 kb fragment corresponding to the episome only in pCam-Pfark-1-KO transfectants. A PCR using primer pair OL7 and OL6 described above amplifying a 1.55 kb fragment corresponding to the wild-type *Pfark-1* locus was used as a control of the DNA samples. Primer pairs OL7/OL168 and OL167/OL6 were used to tentatively amplify across the integration site giving rise to expected 1.25 kb and 1.30 kb products should the *Pfark-1* locus been disrupted respectively.

### Western blotting

Western blot analysis of transfectants and wild-type 3D7 *P. falciparum* parasites was performed on protein extracts prepared by resuspending parasite pellets in M-PER Mammalian Protein Extraction Reagent (Pierce) supplemented with 1 mM phenylmethylsulphonyl fluoride and Complex™ mixture protease inhibitor tablet from Roche Applied Science. The lysates were cleared by centrifugation (15 000 r.p.m. for 15 min at 4°C), and the total amount of proteins in the supernatant was measured by a Bio-Rad protein assay. Ten micrograms of each extract was boiled in Laemmli reducing sample buffer, separated on a 12% SDS-polyacrylamide gel, and subsequently electrotransferred to nitrocellulose membrane (Bio-Rad). The blot was blocked in 5% skim milk and Tris-buffered saline containing 0.05% Tween 20 overnight at 4°C. Immunoblotting was performed using mouse monoclonal anti-GFP antibody (1:5000 dilution) (Roche) and horseradish peroxidase-conjugated sheep anti-mouse IgG antiserum (1:5000 dilution) (Sigma). The membrane was stripped using the Restore™ Western blot Stripping Buffer (Thermo Scientific) according to the manufacturer's protocol, then re-blocked and probed with rabbit anti-*P. falciparum* 2 Cys-Peroxiredoxin antibodies kindly provided by Dr Sylke Müller (WCMP, University of Glasgow, UK).

### Immunoprecipitation and kinase assays

Pfark-1–GFP was immunoprecipitated from parasite protein extracts using the GFP-Trap_A kit (Chromotek). Briefly, protein extracts (∼0.5 mg) in M-PER lysis buffer and protease inhibitors obtained from equal numbers of synchronized schizont stage 3D7 or Pfark-1–GFP transfectant parasites were incubated with anti-GFP-Trap beads for 2 h at 4°C, washed four times with RIPA buffer (Merckx *et al*., 2003) containing 0.5% Triton X-100, 0.5% Nonidet P40 and 0.5% deoxycholate, once with RIPA buffer containing 0.1% SDS and once with kinase buffer (25 mM Tris-HCl, pH 7.5, 15 mM MgCl_2_, 15 mM ATP). Kinase assays were performed in a standard reaction containing kinase buffer supplemented with 5 µCi of [γ-^32^P]-ATP (3000 Ci mmol^−1^, Amersham Biosciences) using immunoprecipitates bound to the anti-GFP beads and 5 µg of substrate, α- and β-casein, or histone H1 and MBP (purchased from Sigma). The reaction proceeded for 30 min at 30°C and was stopped by the addition of Laemmli buffer, boiled for 3 min, and analysed by electrophoresis on 12% SDS-polyacrylamide gel. The gels were dried and submitted to autoradiography.

### Fluorescence microscopy

Imaging was performed on a Delta vision deconvolution fluorescence microscope (100×/1.4 oil immersion objective Olympus IX-70). Images were processed using imaris version 7.0. Expression of the Pfark-1–GFP fusion protein was analysed through direct detection of the green fluorescence in live parasites. Parasite nuclei were stained by incubation 10 min at 37°C in complete medium containing 1 µg ml^−1^ Hoechst 3342 (Invitrogen). For ER staining, the parasite cultures were incubated 30 min at 37°C in complete medium containing 0.5 µM ER-Tracker (Invitrogen). Stained parasites were diluted and placed on a slide and coverslip prior to fluorescence microscopy examination. For immunofluorescence analysis, thin smears of parasite cultures were air-dried and fixed using 4% paraformaldehyde (EMS) in phosphate-buffered saline (PBS) for 20 min at room temperature. Cells were permeabilized with 0.1% Triton X-100 in PBS for 10 min followed by blocking in 3% BSA in PBS overnight at 4°C. The samples were stained by subsequent incubations with a mouse monoclonal antibody specific to chicken brain α-tubulin (clone DM1A, purchased from Sigma) (1:500 dilution), and a secondary TRITC-labelled goat anti-mouse IgG (Southern Biotech, Birmingham, AL) (1:250 dilution) for 1 h at room temperature. All washes used PBS. Slides were stained with DAPI to visualize parasite nuclei and mounted with a DAPCO (Sigma) anti-fading solution.
