# Supplementary material for: An essential Aurora-related kinase transiently associates with spindle pole bodies during Plasmodium falciparum erythrocytic schizogony
Source: Mol Microbiol. 2011 Jan;79(1):205–21. doi: 10.1111/j.1365-2958.2010.07442.x (PMC3025120; doi:10.1111/j.1365-2958.2010.07442.x)
Supplement: Supplementary file 1 [file mmi0079-0205-SD1.pdf]

Supporting information for the article:

An essential Aurora-related kinase transiently associates with spindle pole bodies during *Plasmodium falciparum* erythrocytic schizogony

Luc Reininger, [1*] Jonathan M. Wilkes, [2] Hélène Bourgade, [1] Diego Miranda-Saavedra [3] and Christian Doerig[1]

Table S1 Accession numbers of sequences mentioned in the manuscript.

Table S2 Conserved Ser/Thr residues of Pfark-1 orthologues in Apicomplexa.

Figure S1 Attempts to disrupt the *Pfark-1* gene in *P. falciparum*.

Table S1. Kinase identifiers and annotations

Internal code	UniProt identifier	UniProt annotation
AT10015	O22932	CIPKB_ARATH CBL-interacting serine/threonine-protein kinase 11
AT10457	O22971	CIPKD_ARATH CBL-interacting serine/threonine-protein kinase 13
AT11770	O64629	AUR3_ARATH Serine/threonine-protein kinase Aurora-3
AT12525	Q9CAU7	NEK2_ARATH Serine/threonine-protein kinase Nek2
AT12671	O22042	M3K3_ARATH Mitogen-activated protein kinase kinase kinase 3
AT12910	Q9SFB6	Q9SFB6_ARATH Putative MAP3K epsilon protein kinase
AT12946	Q0V851	Q0V851_ARATH AT3G08720 protein
AT12948	P42818	KPK1_ARATH Serine/threonine-protein kinase AtPK1/AtPK6 OS
AT13159	Q4V3C8	Q4V3C8_ARATH At3g10540
AT13362	Q9LHI7	NEK7_ARATH Serine/threonine-protein kinase Nek7
AT14392	Q9LT35	NEK6_ARATH Serine/threonine-protein kinase Nek6
AT14622	Q9XIW0	CIPK7_ARATH CBL-interacting serine/threonine-protein kinase 7
AT15695	Q0WPH8	NEK5_ARATH Serine/threonine-protein kinase Nek5
AT17	Q9MAM1	CIPK9_ARATH CBL-interacting serine/threonine-protein kinase 9
AT17649	Q94C95	Q94C95_ARATH Putative serine/threonine-protein kinase
AT17791	Q8RXT4	NEK4_ARATH Serine/threonine-protein kinase Nek4
AT21159	O65554	CIPK6_ARATH CBL-interacting serine/threonine-protein kinase 6
AT21386	Q9M077	AUR1_ARATH Serine/threonine-protein kinase Aurora-1
AT22320	P92937	CIPKF_ARATH CBL-interacting serine/threonine-protein kinase 15
AT22608	Q9XF67	Q9XF67_ARATH 3-phosphoinositide-dependent protein kinase-1
AT22889	Q9LYQ8	CIPK2_ARATH CBL-interacting serine/threonine-protein kinase 2
AT23257	Q9LEU7	CIPK5_ARATH CBL-interacting serine/threonine-protein kinase 5
AT24328	Q84VQ3	CIPKQ_ARATH CBL-interacting serine/threonine-protein kinase 26
AT24684	Q8W1D5	CIPKP_ARATH CBL-interacting serine/threonine-protein kinase 25
AT25011	Q8RX66	NEK3_ARATH Serine/threonine-protein kinase Nek3
AT25323	Q9LDI3	CIPKO_ARATH CBL-interacting serine/threonine-protein kinase 24
AT26442	Q9FJ55	CIPKJ_ARATH CBL-interacting serine/threonine-protein kinase 19
AT27841	Q9C562	CIPKA_ARATH CBL-interacting serine/threonine-protein kinase 10
AT28274	Q9LE81	Q9LE81_ARATH IRE OS
AT3163	Q93VD3	CIPKN_ARATH CBL-interacting serine/threonine-protein kinase 23

AT3941	Q9MAJ4	Q9MAJ4_ARATH F27F5.23
AT4295	Q9SX42	Q9SX42_ARATH F14I3.15 protein
AT4769	Q9SLI2	NEK1_ARATH Serine/threonine-protein kinase Nek1
AT6179	O24527	O24527_ARATH Putative serine/threonine kinase
AT9513	Q683C9	AUR2_ARATH Serine/threonine-protein kinase Aurora-2
AT9631	Q2V452	CIPK3_ARATH CBL-interacting serine/threonine-protein kinase 3
DD10749	Q55BN8	NEK2_DICDI Probable serine/threonine-protein kinase nek2
DD11064	Q9U567	Q9U567_DICDI Rac serine/threonine kinase homolog
DD11215	P25323	MYLKA_DICDI Myosin light chain kinase A
DD1129	P34102	PK3_DICDI Protein kinase 3
DD1181	Q54L81	Y0700_DICDI Probable serine/threonine-protein kinase DDB_G0286841
DD12012	Q55FT4	TSUA_DICDI Probable serine/threonine-protein kinase tsuA OS
DD12107	Q869W6	MYLKG_DICDI Probable myosin light chain kinase DDB_G0275057
DD12567	Q54UZ1	Y1639_DICDI Probable serine/threonine-protein kinase DDB_G0280717
DD1535	Q86CS2	ATG1_DICDI Serine/threonine-protein kinase atg1 OS
DD1967	Q76P07	Y7165_DICDI Probable serine/threonine-protein kinase DDB_G0277165
DD260	Q86AD7	MYLKB_DICDI Probable myosin light chain kinase DDB_G0271550
DD3704	Q8T2I8	SEPA_DICDI Serine/threonine-protein kinase sepA
DD3914	Q86HN7	PLK_DICDI Probable serine/threonine-protein kinase PLK
DD4039	Q54QD5	NEK1_DICDI Probable serine/threonine-protein kinase nek1
DD4288	Q54RV3	PAKG_DICDI Serine/threonine-protein kinase pakG
DD4617	Q54HS0	Q54HS0_DICDI Putative uncharacterized protein nek4 (Fragment)
DD5485	Q86I06	NEK3_DICDI Probable serine/threonine-protein kinase nek3
DD6032	P34101	FHKC_DICDI Probable serine/threonine-protein kinase fhkC
DD6168	P28178	PK2_DICDI Protein kinase 2
DD6778	Q54BF0	FHKA_DICDI Probable serine/threonine-protein kinase fhkA
DD7492	P34099	KAPC_DICDI cAMP-dependent protein kinase catalytic subunit
DD7524	Q8MYF1	Y2070_DICDI Probable serine/threonine-protein kinase DDB_G0277449
DD7566	Q54WX4	AURK_DICDI Probable serine/threonine-protein kinase aurK
DD772	Q54VI1	FHKE_DICDI Probable serine/threonine-protein kinase fhkE
DD8277	Q54PK9	PDPKB_DICDI 3-phosphoinositide-dependent protein kinase B
DD8411	Q54CY9	MYLKD_DICDI Probable myosin light chain kinase DDB_G0292624

GL1365	A8B5W4	A8B5W4_GIALA Kinase, NEK
GL1418	A8BNP0	A8BNP0_GIALA Kinase, NEK
GL1454	A8BXP9	A8BXP9_GIALA Kinase, NEK
GL1725	A8BBR0	A8BBR0_GIALA Kinase, NEK
GL1760	A8BDH0	A8BDH0_GIALA Kinase, NEK
GL1829	A8BZJ9	A8BZJ9_GIALA Kinase, NEK
GL2246	A8W2E5	A8W2E5_GIALA cAMP-dependent protein kinase A catalytic subunit
GL2266	A8BME4	A8BME4_GIALA Kinase, NEK
GL3105	A8BQV8	A8BQV8_GIALA Kinase, NEK
GL3389	A8BHW1	A8BHW1_GIALA Kinase, CAMK CAMKL
GL3731	A8BUD9	A8BUD9_GIALA Kinase, NEK
GL3877	A8B882	A8B882_GIALA Kinase, NEK
GL4161	A8BU43	A8BU43_GIALA Kinase, CAMK CAMKL
GL4605	A8BGC2	A8BGC2_GIALA Kinase, NEK
GL592	A8B9E2	A8B9E2_GIALA Kinase, NEK
GL658	A8BDZ5	A8BDZ5_GIALA Kinase, NEK
GL661	A8BBM9	A8BBM9_GIALA Aurora kinase OS
GL665	A8BJT1	A8BJT1_GIALA Kinase, NEK OS
GL7837	A8BVL0	A8BVL0_GIALA Kinase,
GL8038	A8B353	A8B353_GIALA Kinase, NEK
GL9354	A8BLC3	A8BLC3_GIALA Kinase, NEK
GL9611	A8B614	A8B614_GIALA Kinase, NEK
HS11190	Q8TDC3	BRSK1_HUMAN BR serine/threonine-protein kinase 1
HS11253	Q6DLZ0	Q6DLZ0_HUMAN Aurora/Ipl1-related kinase 3 transcript variant 1
HS11590	Q8IVH8	M4K3_HUMAN Mitogen-activated protein kinase kinase kinase kinase 3
HS11620	Q02156	KPCE_HUMAN Protein kinase C epsilon type
HS12613	Q9NRP7	STK36_HUMAN Serine/threonine-protein kinase 36
HS12925	Q9P286	PAK7_HUMAN Serine/threonine-protein kinase PAK 7
HS13178	Q9HBY8	SGK2_HUMAN Serine/threonine-protein kinase Sgk2
HS13315	O14965	STK6_HUMAN Serine/threonine-protein kinase 6
HS14290	Q14012	KCC1A_HUMAN Calcium/calmodulin-dependent protein kinase type 1
HS14423	Q9C098	DCLK3_HUMAN Serine/threonine-protein kinase DCLK3
HS14483	Q9NRH2	SNRK_HUMAN SNF-related serine/threonine-protein kinase
HS14660	P51957	NEK4_HUMAN Serine/threonine-protein kinase Nek4
HS15019	Q8NG66	NEK11_HUMAN Serine/threonine-protein kinase Nek11
HS15979	Q13557	KCC2D_HUMAN Calcium/calmodulin-dependent protein kinase type II subunit delta
HS16032	O00444	PLK4_HUMAN Serine/threonine-protein kinase PLK4
HS16100	Q8N568	DCLK2_HUMAN Serine/threonine-protein kinase DCLK2
HS16178	Q96PY6	NEK1_HUMAN Serine/threonine-protein kinase Nek1
HS16481	Q9NYY3	PLK2_HUMAN Serine/threonine-protein kinase PLK2
HS16709	Q16566	KCC4_HUMAN Calcium/calmodulin-dependent protein kinase type IV
HS16987	A8K161	A8K161_HUMAN Calcium/calmodulin-dependent protein kinase

HS1773	Q8TDX7	NEK7_HUMAN Serine/threonine-protein kinase Nek7
HS18169	O00141	SGK1_HUMAN Serine/threonine-protein kinase Sgk1
HS18324	Q15349	KS6A2_HUMAN Ribosomal protein S6 kinase alpha-2
HS1900	Q96NX5	KCC1G_HUMAN Calcium/calmodulin-dependent protein kinase type 1G
HS1920	P51955	NEK2_HUMAN Serine/threonine-protein kinase Nek2
HS19856	Q96BR1	SGK3_HUMAN Serine/threonine-protein kinase Sgk3
HS20565	P22612	KAPCG_HUMAN cAMP-dependent protein kinase catalytic subunit gamma
HS20889	Q9HC98	NEK6_HUMAN Serine/threonine-protein kinase Nek6
HS21292	P51812	KS6A3_HUMAN Ribosomal protein S6 kinase alpha-3
HS2242	Q04759	KPCT_HUMAN Protein kinase C theta type
HS2265	Q8IU85	KCC1D_HUMAN Calcium/calmodulin-dependent protein kinase type 1D
HS2623	Q13555	KCC2G_HUMAN Calcium/calmodulin-dependent protein kinase type II subunit gamma
HS3291	Q9BYT3	STK33_HUMAN Serine/threonine-protein kinase 33
HS3761	Q7KZI7	MARK2_HUMAN Serine/threonine-protein kinase MARK2
HS390	Q15418	KS6A1_HUMAN Ribosomal protein S6 kinase alpha-1
HS4251	Q9Y2K2	SIK3_HUMAN Serine/threonine-protein kinase SIK3
HS5505	O75385	ULK1_HUMAN Serine/threonine-protein kinase ULK1
HS5633	O15075	DCLK1_HUMAN Serine/threonine-protein kinase DCLK1
HS5742	Q6P3R8	NEK5_HUMAN Serine/threonine-protein kinase Nek5
HS5748	P51956	NEK3_HUMAN Serine/threonine-protein kinase Nek3
HS5833	Q9Y6E0	STK24_HUMAN Serine/threonine-protein kinase 24
HS6389	Q8TD19	NEK9_HUMAN Serine/threonine-protein kinase Nek9
HS6460	O75582	KS6A5_HUMAN Ribosomal protein S6 kinase alpha-5
HS6760	A8K2G2	A8K2G2_HUMAN P21(CDKN1A)-activated kinase 6, isoform CRA_a
HS6972	Q9UIK4	DAPK2_HUMAN Death-associated protein kinase 2
HS8506	Q96GD4	AURKB_HUMAN Serine/threonine-protein kinase 12
HS8644	Q8IYT8	ULK2_HUMAN Serine/threonine-protein kinase ULK2
HS8703	Q86SG6	NEK8_HUMAN Serine/threonine-protein kinase Nek8
HS911	Q16513	PKN2_HUMAN Serine/threonine-protein kinase N2
HS9207	Q4R5I9	Q4R5I9_MACFA QflA-12580, similar to human ribosomal protein S6 kinase
HS9942	A4CYL7	A4CYL7_HUMAN Putative map kinase interacting kinase [Homo sapiens] (Fragment)
PF1135	Q8IEG4	Q8IEG4_PLAF7 Serine/threonine protein kinase (PF13_0085)
PF146	Q8I4W3	Q8I4W3_PLAF7 Rac-beta serine/threonine protein kinase, PfPKB (PFL2250c)
PFC0385c	O77328	O77328_PLAF7 Serine/threonine protein kinase, putative
PF2600	Q9NJU9	CDPK3_PLAF7 Calcium-dependent protein kinase 3 (PFC0420w)
PFL0080c	Q8I629	Q8I629_PLAF7 Serine/threonine-protein kinase, Pfnek-3
PFE1290w	C0H4G2	C0H4G2_PLAF7 Serine/threonine-protein kinase Nek-2
PF4279	Q8ICR0	CDPK2_PLAF7 Calcium-dependent protein kinase 2 (PFF0520w)
PFF0260w	C6KSQ1	C6KSQ1_PLAF7 Serine/threonine protein kinase,

		Pfnek-5
MAL7P1.100	C0H4N8	C0H4N8_PLAF7 Protein kinase
PF4586	Q8IBS5	CDPK4_PLAF7 Calcium-dependent protein kinase 4 (PF07_0072)
PFL1370w	Q8I5D5	Q8I5D5_PLAF7 NIMA-related protein kinase, Pfnek-1
PF536	Q8IDV5	Q8IDV5_PLAF7 Calcium-dependent protein kinase, putative (PF13_0211)
MAL13P1.278	Q8IDD4	Q8IDD4_PLAF7 Serine/threonine protein kinase, putative
TB1184	Q384J7	Q384J7_9TRYP Protein kinase, putative
TB1830	Q57ZK4	Q57ZK4_9TRYP Serine/threonine-protein kinase NEK1, putative
TB2112	Q38CJ2	Q38CJ2_9TRYP Protein kinase, putative
TB254	Q57YR8	Q57YR8_9TRYP Serine/threonine-protein kinase A, putative
TB33	Q57WH3	Q57WH3_9TRYP Protein kinase, putative
TB4147	Q3S1K7	Q3S1K7_TRYBB Protein kinase A-like kinase
TB4373	Q3S1L1	Q3S1L1_TRYBB Protein kinase A-like kinase
TB463	Q57YE4	Q57YE4_9TRYP Serine/threonine kinase, putative
TB4853	Q57V67	Q57V67_9TRYP Serine/threonine-protein kinase, putative
TB5460	Q38B72	Q38B72_9TRYP Protein kinase, putative
TB6126	Q57ZV8	Q57ZV8_9TRYP Protein kinase, putative
TB6454	Q38FY2	Q38FY2_9TRYP Protein kinase, putative
TB6568	Q580Q4	Q580Q4_9TRYP Serine/threonine-protein kinase A, putative
TB7142	Q580F7	Q580F7_9TRYP Serine/threonine-protein kinase, putative
TB7313	Q388G0	Q388G0_9TRYP Protein kinase, putative
TB7601	Q57YQ0	Q57YQ0_9TRYP Serine/threonine-protein kinase NrkA
TB7912	Q580P8	Q580P8_9TRYP Serine/threonine-protein kinase NrkA
TB8391	Q586J4	Q586J4_9TRYP Protein kinase, putative
TB8840	Q388U5	Q388U5_9TRYP Protein kinase A catalytic subunit
TP11023	B8C3Y1	Predicted protein
TP4002	B8C8L4	B8C8L4_THAPS Myosin light chain kinase
TP4762	B8LE83	B8LE83_THAPS Putative uncharacterized protein
TP6606	B8C1H9	B8C1H9_THAPS Predicted protein (Fragment)
TP8741	B5YMU9	B5YMU9_THAPS Predicted protein

Table S2. Pfark-1 orthologues from Apicomplexa do not contain the conserved T^{288} residue within the activation loop of Aurora members.

Phylum	Species	Gene	Aligned residues[a]		
**Metazoa**	Human	aurA	G^{198}	T^{287}	T^{288}
		aurB	N	K	T
**Fungi**	Yeast	Ipl1/Ark1	G	K/T	T
**Apicomplexa**	*P. falciparum*	PFF0260w	T	S	H
	P. vivax	PVX_113435	T	S	H
	P. knowlesi	PKH_114430	T	S	H
	P. berghei	PB000863.02.0	T	S	H
	P. yoelii	Py05614	T	S	H
	P.chabaudi	PCAS_010470	T	S	H
	T. gondii	TGME49_010280	T	S	I
	N. caninum	NCLIV_043570	T	S	I
	B. bovis	XP_001610257-1	T	S	H
	T. annulata	TA11795	T	N	F
	T. parva	TP_764755	T	N	F

[a] Residues present at positions corresponding to residue 198, 287 and 287 of human Aurora A; mutation of G^{198} to N converts human Aurora A to Aurora B-like kinase (Fu et al., 2009).
